# Bacterial Infections Affect Male Fertility: A Focus on the Oxidative Stress-Autophagy Axis

**DOI:** 10.3389/fcell.2021.727812

**Published:** 2021-10-21

**Authors:** Sutian Wang, Kunli Zhang, Yuchang Yao, Jianhao Li, Shoulong Deng

**Affiliations:** ^1^State Key Laboratory of Livestock and Poultry Breeding, Guangdong Key Laboratory of Animal Breeding and Nutrition, Institute of Animal Science, Guangdong Academy of Agricultural Sciences, Guangzhou, China; ^2^Institute of Animal Health, Guangdong Provincial Key Laboratory of Livestock Disease Prevention, Guangdong Academy of Agricultural Sciences, Guangzhou, China; ^3^College of Animal Science and Technology, Northeast Agricultural University, Harbin, China; ^4^Guangdong Laboratory for Lingnan Modern Agriculture, Maoming, China; ^5^Institute of Laboratory Animal Sciences, Chinese Academy of Medical Sciences and Comparative Medicine Center, Peking Union Medical College, Beijing, China

**Keywords:** bacterial infection, male fertility, autophagy, oxidative stress, signal transduction pathway

## Abstract

Numerous factors trigger male infertility, including lifestyle, the environment, health, medical resources and pathogenic microorganism infections. Bacterial infections of the male reproductive system can cause various reproductive diseases. Several male reproductive organs, such as the testicles, have unique immune functions that protect the germ cells from damage. In the reproductive system, immune cells can recognize the pathogen-associated molecular patterns carried by pathogenic microorganisms and activate the host’s innate immune response. Furthermore, bacterial infections can lead to oxidative stress through multiple signaling pathways. Many studies have revealed that oxidative stress serves dual functions: moderate oxidative stress can help clear the invaders and maintain sperm motility, but excessive oxidative stress will induce host damage. Additionally, oxidative stress is always accompanied by autophagy which can also help maintain host homeostasis. Male reproductive system homeostasis disequilibrium can cause inflammation of the genitourinary system, influence spermatogenesis, and even lead to infertility. Here, we focus on the effect of oxidative stress and autophagy on bacterial infection in the male reproductive system, and we also explore the crosslink between oxidative stress and autophagy during this process.

## Introduction

Healthy fertility is the basis for the survival and continuation of the species. Recently, however, there have been increasing reports of declining male fertility. It is estimated that infertility affects hundreds of millions of reproductive-aged couples worldwide (Inhorn and Patrizio, 2015). Approximately 50% of these infertility cases are caused by male infertility (Vander Borght and Wyns, 2018). The factors affecting male fertility include genetic defects (Brugh and Lipshultz, 2004; Ferlin et al., 2006), steroid hormone disorders (O’Hara and Smith, 2015), hypogonadism (Casarini et al., 2020), spermatogenesis dysfunction (Gunes et al., 2015), ejaculation disorders (Fode et al., 2012) and reproductive infections (Reddy et al., 2006; Ochsendorf, 2008; Sarkar et al., 2011). The incidence of infertility varies significantly in different countries, and regions are very different. In developed countries, about 10% of male infertility is related to infectious and immune factors (Bachir and Jarvi, 2014). In developing countries with poor health and medical conditions, this proportion is as high as 50% (Ekwere, 1995). These differences could be regarded as sanitary condition, lifestyle, religious faith and medical resource. Pathogenic microorganism infection and its induced immune response are important causes of male infertility. As particular immune organs, the testis and epididymis protect sperm from adverse immune responses and effectively resist pathogenic microbial infection (Fijak et al., 2011).

Various microorganisms, namely bacteria, viruses, and parasites, can infect the male reproductive system and induce a series of inflammatory responses that impair male fertility (Bukharin et al., 2000). Bacteria usually infect the urethra, seminal vesicles, prostate, epididymis, vas deferens, and testes retrograde through the reproductive tract. Infections caused by bacteria, including *Chlamydia trachomatis*, *Neisseria gonorrhoeae*, and Brucella, contribute to 15% of male infertility cases (Schaeffer, 1998; La Vignera et al., 2011; Cai et al., 2014; Erdem et al., 2014). Furthermore, Mycoplasma can infect the male reproductive tract but does not affect male fertility. However, it can be transmitted to females and impair female fertility by sex. Here we summarize the effect of bacterial infections on the male internal reproductive system and explore the underlying mechanisms.

After the pathogenic bacteria invade the male reproductive system, oxidative stress and autophagy could be induced by cells in the gonads. Moderate oxidative stress helps clear the pathogen, but excessive oxidative stress can induce testicular damage or even lead to infertility (Marchlewicz et al., 2016; Huo et al., 2019). Abnormally elevated oxidative stress has a toxic effect on tissue and cells, and can damage almost everything inside cells, including DNA, proteins, and lipids. Autophagy is a highly conserved cytological behavior in all eukaryotes that maintains homeostasis by breaking down intracellular proteins and organelles to provide energy and metabolic raw material. Increasing evidence suggests that autophagy takes part in a series of events within the male reproductive system, including spermatogenesis, and hormone metabolism, which are also affected by oxidative stress (Sharma et al., 2019; Zhu et al., 2019). Coincidentally, autophagy, which is always accompanied by oxidative stress, helps maintain immune homeostasis (Li et al., 2019). Therefore, it is of positive significance to explore the effect of oxidative stress-autophagy axis on the male reproductive system. In this review, we focus on exploring their functions on the male reproductive system during bacterial infections.

## Male Infertility and Bacterial Infections

The effect of bacterial infection on reproductive system function is important. Various bacteria have been isolated and identified from the male reproductive system, including *Escherichia coli*, *Staphylococcus aureus*, *Ureaplasma urealyticum*, *C. trachomatis*, *N. gonorrhoeae*, *Streptococcus agalactia*, and *Staphylococcus saprophyticus* (Oghbaei et al., 2020). These bacteria lead to all kinds of diseases, such as chlamydiosis, gonorrhea, and ureaplasmosis, which can cause male reproductive system infections (Trojian et al., 2009). Bacterial infection induces male infertility in the following ways (Table 1).

**TABLE 1:** Common bacterial infection and its effect on male fertility.

Pathogenic bacteria	Sites of infection	Effect on fertility
*C. trachomatis*	Testis, epididymis, seminal vesicle, urethra, prostate	Spermatogenesis, sperm motility and morphology, sperm DNA damage, orchitis
*N. gonorrhoeae*	Testis, epididymis, seminal vesicle, urethra	Spermatogenesis, sperm DNA damage, orchitis
*U. urealyticum*	Epididymis, urethra, prostate	Sperm motility and morphology, inflammation, sperm DNA damage, orchitis
*P. aeruginosa*	Testis, epididymis, urethra	Spermatogenesis, sperm DNA damage, orchitis
*E. coli*	Testis, epididymis, seminal vesicle, urethra, prostate	Spermatogenesis, sperm motility and morphology, sperm DNA damage, orchitis
*S. aureus*	Epididymis, urethra	Sperm motility and morphology, sperm DNA damage, orchitis
*Brucella*	Testis, epididymis, seminal vesicle	Orchitis

### Bacterial Infections Affects Male Fertility

*Chlamydia trachomatis*, an intracellular bacterium, has been detected in the epididymis, urethra, prostate, and Leydig cells of the testis (Cunningham and Beagley, 2008). Although *C. trachomatis* is a non-motile bacterium, it can infect testicular cell populations (sperm, Leydig, and Sertoli cells) in only 3 days by “hijacking” testicular macrophages (Bryan et al., 2019). However, it is not clear how infected macrophages transfer infection to other cells in the testes. There is evidence that LPS of *C. trachomatis* interacts with the CD14 on the sperm surface, and then induces production of oxidation intermediates, sperm membrane lipid peroxidation, sperm DNA damage and caspase-mediated apoptosis (Satta et al., 2006). *C. trachomatis* infection-induced deterioration of sperm quality was associated with a low potential for male fecundity (Gallegos et al., 2008).

*Neisseria gonorrhoeae* causes the most common infectious diseases in men reproductive system (Furuya and Tanaka, 2009). It causes urethritis, prostatitis, epididymitis, and are accompanied by the secretion of mucopurulent urethral discharge. Available studies found a detrimental effect of *N. gonorrhoeae* on male fertility (Abusarah et al., 2013). *N. gonorrhoeae* can attach to the spermatozoa by its pili and then infects other tissues through triggers a flow of polymorphonuclear leukocytes (Stohl et al., 2012). There are very few studies that reported the relationship between *N. gonorrhoeae* and male infertility. The studies only reported *N. gonorrhoeae* were twice as common in the semen of infertile patients (Khoder et al., 2019). The molecular mechanism of *N. gonorrhoeae* infection-induced abnormal sperm and reduction in sperm fertilization rate remains unclear. It is noteworthy that *N. gonorrhoeae* can turn over a large amount of peptidoglycan and is capable of activating Toll-like receptors (TLRs) and Nod-like receptors (NLRs) to trigger antibacterial innate immunity (Mavrogiorgos et al., 2014). Furthermore, *N. gonorrhoeae* infection can induce apoptosis and oxidation intermediates production in semen via promoting the production of IL-1β (Kemal Duru et al., 2000; Singer and Ouburg, 2016).

*Ureaplasma urealyticum* is responsible for male infertility and is implicated in the pathogenesis of epididymitis, prostatitis, and urethritis (Pellati et al., 2008). Adhesion of *U. urealyticum* to the sperm decreases sperm motility but the exact mechanism by which *U. urealyticum* affects sperm quality has not yet been revealed (Nunez-Calonge et al., 1998). Interestingly, metabolic products of *U. urealyticum*, such as H_2_O_2_ and OH−, are toxic to the sperm (Farsimadan and Motamedifar, 2020). So some researchers thought that *U. urealyticum* in the urethra make sperm more vulnerable to peroxidation damage and infertility (Shang et al., 1999). Controversially, other researchers claimed that *U. urealyticum* infection did not cause changes in sperm motility, concentration, morphology and viability (Gdoura et al., 2007). We speculated that the differences in the results of different studies might be caused by the time/dose of infection and the cross-infection of multiple bacteria.

*Pseudomonas aeruginosa* is a frequent inducer of orchitis, epididymitis and urethritis (Rana et al., 2018). *P. aeruginosa* is less toxic than other bacteria, which triggers chronic infections by eliciting low levels of inflammatory responses (Farsimadan and Motamedifar, 2020). Exotoxin A of *P. aeruginosa* targets sperm tail proteins and affects sperm motility (Farsimadan and Motamedifar, 2020). Porin from *P. aeruginosa* has sperm plasma membrane receptors, directly affecting sperm parameters, such as inducing apoptosis of seminal vesicle epithelial cells (Buommino et al., 1999).

Brucella can survive and replicate in immune cells by escaping and regulating the host immune response, and spread to the tissues where Brucella is susceptible to colonization through cell chemotaxis (Barquero-Calvo et al., 2007; Martirosyan et al., 2011). However, Brucella has a non-classical LPS different from the classical LPS of other bacteria, such as *E. coli*, and only leads to a weaker inflammatory response (Rossetti et al., 2012). In addition, Brucella also can inhibit the maturation of phagolysosome and then migrate to the endoplasmic reticulum and fuse with it to ensure its survival (Celli et al., 2003). Subsequently, as these infected bacteria proliferate in the male reproductive system, they can induce cytokines and produce a state of inflammation such as orchitis and epididymitis.

*Escherichia coli* is the most significant bacterium in bacterial infection-mediated male infertility (Comhaire et al., 1999). *E. coli* -induced male infertility is multifaceted, including inflammation of genitourinary system, failure of steroidogenesis and spermatogenesis, and deteriorating sperm quality. Components extracted from *E. coli*, such as LPS and porins, can bind to cells’ receptors in the gonads and cause inflammatory responses, oxidative stress and apoptosis via various signals (Galdiero et al., 1988). Accumulation of proinflammatory cytokines and oxidative intermediates eventually leads to male infertility (Zeyad et al., 2018b). *E. coli* LPS is related to the activation of NF-κB, HIF-1α signaling and inflammatory responses (Palladino et al., 2018).

### Effect of Bacterial Infections on the Male Reproductive Organs

The maintenance of the male reproductive function depends on effective spermatogenesis and the synthesis of testosterone. Tissue damage and inflammation caused by bacterial infection can lead to male infertility by negatively affecting spermatogenesis and testosterone synthesis (Schuppe et al., 2008). Inflammation is the body’s defense mechanism against infection. Inflammation responses prompt leukocytes to move to the infection site and clear the infection. In this process, various cytokines such as tumor necrosis factors and interleukins can mediate inflammation to affect testes and epididymis. Moreover, TNF-α, IL1β, and IL-6 can inhibit Leydig cell’s synthesis of testosterone and induce apoptosis of spermatogenic cells. In addition, inflammation is associated with oxidative stress (Agarwal and Saleh, 2002; Reddy et al., 2006; Agarwal et al., 2014). Excessive oxidative stress is linked to male reproductive organ injury and male infertility (Trojian et al., 2009).

### Effect of Bacterial Infections on Cells in the Male Gonads

Some bacterial infections influence spermatogenesis and even lead to sperm apoptosis, which causes male infertility (Rana et al., 2018; Klein et al., 2020). Successful reproduction requires functioning germ cells. Bacterial infection can cause considerable damage to sperm, such as chromosome breakage, change of cell membrane structure, acrosome injury, and mitochondrial dysfunction (Jendrossek et al., 2001; Fraczek et al., 2007, 2012; Haines et al., 2013; Li J. et al., 2018; Zeyad et al., 2018a). The integrity of sperm DNA and chromatin are important factors affecting male fertility. Studies show that damaged DNA harms male fertility (Santi et al., 2017; Simon et al., 2017). Research has shown that *Chlamydia* infections cause sperm chromosome breakage (Bryan et al., 2019). Sertoli, Leydig, and spermatogonial stem cells are essential for normal spermatogenesis. Sertoli cells usually act as guardians for sperm and provide growth factors, nutrients and energy for sperm. On the one hand, a certain number of Sertoli cells are the basis for maintaining spermatogenesis (Sharpe et al., 1999). On the other hand, Sertoli cells are important targets of hormone signal transduction (especially cholesterol), and its abnormal metabolism can lead to disorder of spermatogenesis and ultimately male infertility (Golden et al., 1999). In addition, Sertoli cells can take up and clear apoptotic spermatogonial cells to maintain functioning spermatogenesis. However, Sertoli cells can also be infected through the phagocytosis of infected apoptotic spermatogonial cells (Bryan et al., 2019). Leydig cells are the primary cells for steroid synthesis involved in spermatogenesis, sexual development, maintenance of secondary sexual characteristics and sexual behavior. Inflammation and apoptosis of Leydig cells can cause abnormal testosterone synthesis, which affects spermatogenesis and male reproduction (Theas, 2018). Furthermore, Leydig cells and testicular macrophages are adjacent; thus, cell-to-cell contact can spread the infection (Hales, 2002). Dysfunctions of these cells attributed to bacterial infection can further negatively affect spermatogenesis. The *E. coli*, *S. aureus*, *Mycoplasma*, and *P. aeruginosa* secretion of inflammatory cytokines induced by these bacterial infections also negatively affect spermatogenesis (Said et al., 2005; Fraczek et al., 2013). Moreover, some bacteria, such as *Mycoplasma genitalium*, can infect the male reproductive system without affecting male fertility. However, these bacteria can be transmitted to females by sex and influence female fertility (Horner and Martin, 2017).

## Effects of Oxidative Stress on Male Fertility During Bacterial Infection

Oxidative stress is a state of imbalance between oxidation and antioxidation in favor of the oxidants (Sies, 2015). Forms of Oxidative stress include nutritional oxidative stress, physiological oxidative stress, photooxidative stress, radiation-induced nitrosative stress, and reductive stress. Generally, physiological oxidative stress leads to inflammatory infiltration of neutrophils, increased protease secretion, mitochondrial dysfunction, lipid peroxidation, and the production of many oxidative intermediates such as reactive oxygen species (ROS) and reactive oxygen nitrogen species (RNS), and various cytokines (Ryan et al., 2004). One of the bacterial genitourinary system infection results is the overproduction of ROS and RNS. There is evidence that a low level of ROS/RNS participates in eliminating intracellular bacteria (West et al., 2011). Meanwhile, ROS/RNS and oxidative stresses help eliminate the infections and fertilize sperm. However, excessive ROS/RNS leads to dysregulation of the endogenous ROS/RNS clearance system and induces intense oxidative stress, one of the causes of infertility (Aitken et al., 1989; Pizzino et al., 2017; Borrelli et al., 2018). It has been reported that various bacteria, including *E. coli, Staphylococcus haemolyticus, Bacteroides ureolyticus*, and *C. trachomatis* can cause oxidative stress in different male reproductive systems (Appasamy et al., 2007; Fraczek et al., 2007; Gonzalez-Marin et al., 2011; Neagu et al., 2011). In addition, pathogen-associated molecular patterns of these bacteria, such as LPS and lipoteichoic acid also caused oxidative stress. For example, stimulating Sertoli cells with LPS increases lipid peroxidation and hydrogen peroxide levels, and inhibits the synthesis of antioxidant enzyme activities and glutathione-*S*-transferase. Furthermore, several Sertoli cell function markers, including lactate, lactic acid dehydrogenase, γ-glutamyl transpeptidase, and *b*-glucuronidase levels, were decreased in a dose-dependent manner that affected Sertoli cell’s ability to maintain normal reproduction (Aly et al., 2010). These results show that bacterial infections affect redox equilibrium in the male reproductive system.

### Oxidative Stress and Its Signal Transduction in Bacterial Infections

Under normal physiological conditions, the oxidation intermediates can be promptly removed by various antioxidants, including dismutase (SOD), glutathione reductase, and vitamin E. The remaining oxidation intermediates, such as ROS, can participate in membrane receptor-mediated signal transduction and vascular tension maintenance. However, when the body is subjected to threatening stimuli, oxidation intermediates’ production is too high to be eliminated, and oxidative stress occurs. Multiple oxidation and antioxidation signaling pathways are involved in the process of oxidative stress, including Nrf2/Keap1/antioxidant response elements (AREs) signaling, PI3K/Akt/mTOR signaling, and TLRs signaling.

#### NF-E2-Related Factor/Kelch-Like ECH-Associated Protein 1/Antioxidant Response Elements Signaling

NF-E2-related factor 2 (Nrf2)-Kelch-like ECH-associated protein 1 (Keap1) signaling is a defense system that maintains physiological homeostasis in mammals. In its inactive state, Nrf2 is present in the form of the Nrf2-Keap1 complex in the cytoplasm. When oxidative stress occurs, Nrf2 dissociates from Keap1 and then goes into an activation status and translocates to the nucleus. Dietz found that changes in the structure of the Keap1 protein induced by stimulation through the modification of cysteine residues lead to the dissociation of Nrf2 (Dietz et al., 2008). However, another piece of evidence shows that oxidative stress activates PI3K and mitogen-activated protein kinase (MAPK) signaling and causes the phosphorylation of Nrf2, leading to its dissociation from Keap1 (Tkachev et al., 2011). Subsequently, the free Nrf2 heterodimerizes with musculoaponeurotic fibrosarcoma oncogene homolog (Maf) protein. Then, the Nrf2-Maf heterodimer binds to AREs to induce redox-balancing factors, antioxidants, stress response proteins, and metabolic genes such as *HO-1*, *NQO-1*, *GCL*, *GST*, *GPx*, *SOD*, and *CAT* (Hahn et al., 2015; Yang et al., 2016; Fuse and Kobayashi, 2017). Most studies on Nrf2 signaling focus on tumorigenesis, metabolic disease, and toxic chemical stressors (Higgins et al., 2009; Sporn and Liby, 2012; Nezu et al., 2017). Evidence suggests that *Helicobacter pylori* stimulation can downregulate Nrf2 through NADPH oxidases 1 (Perez et al., 2017). NADPH oxidase 2-deficient mice are susceptible to *S. aureus* and *Burkholderia cepacia* (Pizzolla et al., 2012).

#### PI3K/Akt/mTOR Signaling

PI3K/Akt/mTOR signaling, as a bridge between extracellular signals and intracellular responses, widely exists in various cells and participates in cell growth, proliferation, and differentiation. PI3K/Akt/mTOR signaling is involved in various physiological and pathological processes such as tumorigenesis, pathogenic microbial infection, and autoimmune disease (Mistry et al., 2019; Noorolyai et al., 2019; Jeddi et al., 2021). After PI3K activation, the second messenger PIP3 can combine with Akt and phosphoinositol-dependent protein kinase (PDK). PDK catalyzes the phosphorylation of Akt at Ser308 and Ser473 and leads to complete activation of Akt (Kilic et al., 2017). Activated Akt can mediate apoptosis, cell migration, and autophagy by activating or inhibiting MDM2, Palladin, and mTOR (Hers et al., 2011). Several studies have found that PI3K signaling is closely related to bacterial infection-mediated oxidative stress. *Salmonella typhimurium* infection increases oxidative stress levels, influencing mitochondrial translocation through PI3K/Akt/mTOR signaling (Mistry et al., 2019). A proteomic approach coupled with bioinformatics analysis showed that *Klebsiella pneumoniae* infection causes the misfolding of host proteins through PI3K/Akt/mTOR signaling, and inhibition of mTOR induces autophagy and intestinal atrophy (Kamaladevi and Balamurugan, 2017). Oliveira et al. (2018) confirmed that ROS contributes to the invasion of host cells by *S. agalactia*, with cytoskeletal recombination through PI3K signaling. In addition, mTOR plays an essential role in maintaining male reproduction, and a study showed that mTOR deficiency reduces sperm motility (Schell et al., 2016).

#### Toll-Like Receptors Signaling

The innate immune system consists of various components that coordinate to suppress infection and eliminate invading pathogenic microorganisms. In the past few decades, there has been much evidence that exogenous pathogens invade hosts based on gene-encoded pathogen-associated molecular patterns (PAMPs), and the protective molecules that can recognize these exogenous pathogens are called pattern recognition receptors (PRRs). TLRs are one of the PRR families that can recognize various PAMPs, and TLR2 and TLR4 are the most important PRRs that mainly recognize LPS, lipoteichoic acid, lipoprotein, and lipopeptides in the process of bacterial infections (Akira et al., 2001; Janeway and Medzhitov, 2002). TLR signaling is divided into two pathways; a Myd88-dependent pathway and a TRIF-dependent pathway. In Myd88-dependent pathways, MyD88 first recruits and activates interleukin receptor-associated kinase (IRAK), and then activated IRAK1 combines with TRAF6 to activate TAK1, which in turn induces NF-κB and MAPK signaling pathways to produce inflammatory responses (Kollewe et al., 2004; Akira et al., 2006; Kawai and Akira, 2010; Chen, 2012). In the TRIF-dependent pathway, MyD88 is not necessary. After TRIF is recruited to TLRs, it helps induce inflammatory responses and type I interferon through activation of NF-κB signaling, MAPK signaling, and interferon regulatory factor 3 (Fitzgerald et al., 2003; Guo and Cheng, 2007). The cells are activated by a cascade of signals induced by TLRs, and the activated cells produce pro-inflammatory cytokines that induce the production of ROS and RNS and cause oxidative stress (Ryan et al., 2004). In addition, interferon (IFN) also regulates oxidative stress. IFN promotes hydrogen peroxide release by activating macrophages and can interact with nicotinamide adenine dinucleotide phosphate oxidases 1 and induce superoxide anion production. Furthermore, NF-κB and MAPK signaling can regulate the transcriptional level of inducible nitric oxide synthase, which induces excess NO release and causes oxidative stress (Reimann et al., 1994; Taylor et al., 1998). Additionally, oxidative stress can affect TLR-mediated inflammatory responses. A study found that ROS leads to AP-1 transcriptional activity attenuation, which reduces the transcriptional expression of TLR4 (Ishida et al., 2002).

### Oxidative Stress-Induced Male Infertility in Bacterial Infections

Many factors lead to male infertility, including hormonal disorders, obesity, stress, lifestyle, hygienic conditions, and general health. The male reproductive system infection, especially bacterial infections, is a common factor that impairs male reproductive tract function and spermatogenesis and is a substantial reason for male infertility. Pathogenic bacteria in the male reproductive tract are mainly concerned with genitourinary system dysfunction, failure of steroidogenesis and spermatogenesis, and deteriorating sperm quality, leading to male infertility (Fijak et al., 2018; Oghbaei et al., 2020; de Oliveira et al., 2021). The main consequences of bacterial infection-induced genitourinary system dysfunction, failure of steroidogenesis and spermatogenesis and deterioration of sperm quality are the overproduction of pro-inflammatory cytokines and oxidative stress. For instance, S*taphylococcus* can invade the male reproductive system directly or through blood-borne transmission. *Staphylococcus* affects male fertility, yielding poor-quality semen, increased tissue damage, and impaired sperm functions by releasing pro-inflammatory cytokines and ROS. Staphylococcal exotoxins can also activate T-helper (Th) cells, Th1 and Th17, which aggravates the damage of male reproductive tissue/cells (Dutta et al., 2020). *C. trachomatis* is the most common sexually transmitted bacterium which impairs male fertility by causing urethritis, prostatitis, epididymitis, and orchitis. In addition, it has been shown that *C. trachomatis*-induced the secretion of various cytokines and production of ROS are important causes of these diseases (Witkin et al., 1995; O’Connell et al., 2006).

#### Oxidative Stress Affects Male Reproductive Organ Function

The male reproductive system is mainly composed of testis and accessory organs. Oxidative stress can affect male fertility through damage to male reproductive system. *N. gonorrhoeae* can cause orchitis and epididymitis, which lead to the male reproductive tract injury and obstruction through inflammation and oxidative stress (Ochsendorf, 2008; Mavrogiorgos et al., 2014). Since testicles are mainly responsible for spermatogenesis and androgen secretion. When male infertility occurs, we often consider whether the physiological function of the testis is normal firstly. Excessive oxidative stress induces the continual accumulation of lipid peroxide and consumption of antioxidant enzymes which leads to apoptosis of androgone and Leydig cells and testicular dysfunction (Strycharz et al., 2018). The epididymis is one of the important organs of the male reproductive system and is related to sperm maturation, transport and storage. When the sperm has just left the testes, it is still immature and lacks self-defense mechanisms. Sperm will gain the ability to fertilize only after their descent and maturation within the epididymis. Although a given oxidation level is required for sperm maturation in the epididymis, sperm are sensitive to oxidative damage. Redox balance in the epididymis is the basis of maintaining normal epididymis function. Excessive oxidative stress disrupts the activity of proteins secreted by epididymal epithelium, interferes with sperm plasma membrane fluidity and DNA integrity and ultimately leads to male infertility (Noblanc et al., 2012).

#### Oxidative Stress Affects Male Reproductive Cells Function

When pathogenic bacteria invade the male reproductive system, various cells in the gonads respond to bacteria, including testicular macrophages, Sertoli, and Leydig cells (Chen et al., 2016). In the process, the TLR family contains the most important PRRs, which can recognize these bacteria and cause the production of multiple cytokines and immune responses. For example, *C. trachomatis* can be recognized by TLR2 and TLR4, which induce IL-1β, IL-8, IL-10, IL-17A, IFN-γ, and iNOS in Sertoli cells and testicular macrophages (Winnall et al., 2011; Murthy et al., 2018). In addition, chlamydial LPS, a ligand of TLR4, can cause sperm dysfunction and apoptosis (Hosseinzadeh et al., 2001; Bryan et al., 2020). Moreover, our lab’s studies have revealed that TLRs’ expression influences the production of pro-inflammatory cytokines and the level of oxidative stress (Deng et al., 2020; Wang et al., 2020). Alternatively, bacterial endotoxin lipopolysaccharide-induced ROS can inhibit Leydig cell steroidogenesis and cause Sertoli cell apoptosis (Le Goffic et al., 2003; Zhang et al., 2020). Cumulative oxidative damage may inhibit steroid synthesis in Leydig cells, and abnormal mitochondria may also be associated with Leydig cell apoptosis, leading to decreased sex steroid hormones. In addition, the decline of sexual steroid hormones can aggravate mitochondrial dysfunction, further promote mitochondrial damage, and ultimately aggravate apoptosis (Miller, 2013).

#### Oxidative Stress Affects Sperm Quality and Spermatogenesis

A certain number of functional sperm is the basis of male reproductive function. In the macrophages of *Treponema pallidum-*infected men, high cytokine levels such as IFN-β, IFN-γ, and TNF-α are associated with oxidative stress-induced sperm DNA damage and apoptosis (Cruz et al., 2012; Azenabor et al., 2015). A recent study found that Mycoplasma infection can induce oxidative and mitochondrial dysfunction by activating NF-κB and Nrf2/HO-1 signaling (Ishfaq et al., 2019). On the one hand, excessive oxidative stress leads to the release of mitochondrial substances such as cytochrome C, which activates the caspases signal and induces apoptosis (Wagner et al., 2018). On the other hand, excessive ROS leads to a decrease in mitochondrial membrane potential, which leads to energy generation disorder and ultimately further decreases sperm motility. A high ROS level can affect the fluidity of the sperm membrane and induce sperm mitochondria to produce a high level of lipid peroxidation and even lead to apoptosis (Aitken et al., 2012).

Furthermore, excessive oxidative stress leads to sperm DNA damage, an important factor that induces male infertility. Also, oxidative stress is a double-edged sword in spermatogenesis (Sharma et al., 2019). Since oxidative stress can affect the microenvironment in which spermatogenesis occurs, the negative effects of oxidative stress on spermatogenesis are indisputable (Tremellen, 2008). In addition, excessive oxidative stress induces apoptosis of Sertoli cells and further disrupts spermatogenesis (Sharpe et al., 1999). Moreover, oxidative stress affects spermatogenesis through impairing epigenetics, such as DNA methylation (Sharma et al., 2019).

## Oxidative Stress-Autophagy Axis in Male Fertility

### Autophagy Is an Important Factor Affecting Male Reproductive Homeostasis During Pathogen Infection

Autophagy is a highly conserved cytological behavior in all eukaryotes that maintains homeostasis by breaking down intracellular proteins and organelles. Furthermore, autophagy is a fundamental cell biological pathway that can influence immunity. Autophagy controls inflammation by interacting with innate immune regulatory signals to clear pro-inflammatory cytokines and redundant oxidation intermediates. It is considered that IKK, TAB2/3, mTOR, MAPK, and TAK signaling regulate autophagy. When immune cells recognize the PAMPs from a pathogenic microorganism, Tab2/3 dissociates from Beclin-1 and induces autophagy initiation and autophagosome formation (Monkkonen and Debnath, 2018). Autophagy can be used as an immune barrier to eliminate infectious pathogens, while some pathogens can use autophagy to promote their survival in host cells, thus aggravating infection (Engstrom et al., 2019). Also, excessive autophagy can inhibit the proliferation of spermatogonial, cause seminiferous tubules injury, trigger spermatogenesis dysfunction and even sperm apoptosis (Liu et al., 2017; Mu et al., 2017). Therefore, autophagy is more like a “double-edged sword” in the process of anti-pathogen infection. There is accumulating evidence indicating that autophagy is involved in several pathological and physiological processes in the male reproductive system, including spermatogenesis, testicular endocrinology, fertilization (Zhu et al., 2019). PDGFR-β siRNA-PEI-PLGA-PEG nanoparticles-induced autophagy helps decrease the *C. trachomatis* by approximately 65%. The knocking down of PDGFR-β and promoting autophagic flux in host cells contribute to fighting against *C. trachomatis* (Yang et al., 2019). An interesting study found that CD46-cyt1/GOPC signal-dependent autophagy can reduce the number of *N. gonorrhoeae* invading cells at the early stages of infection (at 2–4 h). Nevertheless, *N. gonorrhoeae* starts to remodel lysosomes and prevent degradation of autophagolysosomal contents, which cause bacteria to survive in it (Kim et al., 2019). T3SS of *P. aeruginosa* inhibits the autophagy process. Thus rapamycin-inducing autophagy could enhance the clearance of *P. aeruginosa* (Xu J. et al., 2020). Moreover, rapamycin-inducing autophagy suppresses *P. aeruginosa*-induced apoptosis and ROS accumulation via MAPK signal and ultimately eliminate bacteria (Han et al., 2020). In addition, autophagy-related gene (ATG) families regulate autophagy and are considered in the cytoskeleton maintenance (Offei et al., 2018). The deletion mutation of ATG5/7 in Leydig cells results in an abnormal accumulation of PDLIM1 and then sperm with malformed heads and low motility (Liu et al., 2016).

Furthermore, autophagy can also affect lipid metabolism, and this process is known as lipophagy. In Leydig cells, inhibition of autophagy leads to a drop in testosterone and free cholesterol. Further research found that autophagy causes the accumulation of NHERF2 and down-regulation of SRBI, leading to inadequate cholesterol intake and decreased testosterone synthesis (Gao et al., 2018; Ma et al., 2018). Moreover, mitophagy helps clear the sperm mitochondrial DNA, potentially toxic to organisms in the normal fertilization course (Sato and Sato, 2011). Deletions in Parkin and MUL1 cause those paternal mitochondria to remain in the embryos (Rojansky et al., 2016). Additionally, autophagy is controlled by various PRRs and responds to PAMPs. In the process of pathogenic infection, bacterial PAMPs activate multiple signaling pathways, such as the NF-κB, MAPK, and PI3K signaling pathways, to induce autophagy (Deretic et al., 2013). These pieces of evidence suggest that autophagy is essential to maintain male fertility.

### Effect of Oxidative Stress-Autophagy Interactions on Male Fertility in Bacterial Infection

Various PRRs in all kinds of immune cells can recognize pathogenic bacteria, and these cells start to eliminate the bacteria by a series of immune responses. ROS is one of the crucial signaling molecules in the oxidative stress response. Under the pathological condition of bacterial infection, low ROS levels can help clear the pathogenic bacterium, but the excessive accumulation of ROS could affect cellular homeostasis, causing oxidative stress and cell dysfunction, and even cell death. Meanwhile, autophagy is usually activated during this process. It suggests that there is a close connection between oxidative stress and autophagy. Evidence shows that oxidative intermediates are the upstream modulators of autophagy (Filomeni et al., 2010). Predictably, appropriate oxidative stress acts as special ‘alarm molecules’ of bacterial infections by signaling their invasion to the autophagic machinery. In turn, moderate autophagy helps maintain physiological homeostasis through a negative feedback regulation by concomitantly reducing ROS and oxidative damage to organelles and ultimately removing bacteria (Kim et al., 2017). Autophagic disorders have been found to be associated with the initiation of pathological states. In the epithelial cells, defects of autophagy-related genes are related to higher cellular ROS levels (Saxena et al., 2018). Moreover, deletions of autophagic genes result in the accumulation of damaged organelles and DNA that induce metabolic disturbance (Larabi et al., 2020). Additionally, there is also evidence that excessive autophagy leads to the aggravation of oxidative damage of testis (Tian et al., 2020). Inhibiting the production of oxidative stress, in turn, contributes to the inhibition of bacterial LPS-induced autophagy (Yuan et al., 2009). From this perspective, autophagy is essential for the male reproductive system to eliminate bacterial infections and oxidative stress status simultaneously. It can be seen from the above that there is a complex relationship between autophagy and oxidative stress. And there is no doubt that the regulation of the oxidative stress-autophagy axis is involved in multiple signaling pathways.

#### Toll-Like Receptors-NF-κB/MAPK Signaling in Oxidative Stress-Autophagy Axis

Various bacteria, isolated and identified from the male reproductive system, such as *C. trachomatis*, *N. gonorrhoeae*, *E. coli*, and *S. aureus*, can be recognized by TLRs and activate the NF-κB signaling pathway, which induces the production of multiple inflammatory cytokines. TNF-α and IL-1 can strongly induce ROS and invoke oxidative stress through the NOX family proteins pathway (Park et al., 2006). Knockdown of Nox4 decreases the LPS-induced ROS generation. The mechanism is that the cytokines-activated Nox enzymes catalyze the conversion of O_2_ to O_2__–_, and then converted to H_2_O_2_ by superoxide dimutase. Moreover, H_2_O_2_-induced oxidative stress can upregulate p62 and increase autophagy by mediating NF-κB p65 phosphorylation at Ser-536 (Song et al., 2017). NF-κB-p62 signals establish the connection between oxidative stress and autophagy. Alternatively, NF-κB promotes the clearing of the damage by activating the autophagy receptor P62 and inhibiting the production of IL-1 via NLRP3 (Zhong et al., 2016). Also, bacterial LPS-mediated activation of TLRs could induce MAPK pathway through TAK1 signaling, inhibition of MAPK signaling causes autophagic dysfunction. Research findings show that p38 MAPK, ERK, and JNK are all involved in the induction of autophagy (Xu et al., 2016; Li Q. et al., 2018; Wang et al., 2020). Activation of MAPK can increase beclin-1 activity, and then regulates transcription of ATG family which can induce autophagy initiation (Zhou et al., 2015). ERK signaling is associated with many autophagic markers. Activation of ERK signal induces the conversion of LC3-I to LC3-II, induction of Beclin-1 and BNIP, and phosphorylation of G-interacting protein and p53 (Ogier-Denis et al., 2000; An et al., 2006; Cheng et al., 2008). Similarly, JNK can mediate the accumulation of p62 and phosphorylation of AMPK, which are involved in the induction of autophagy (Zhou et al., 2015). Moreover, the JNK signal contributes to up-regulating ATG5 and ATG7, the important proteins in the autophagy complex (Wong et al., 2010; Xie et al., 2011). In turn, ROS scavenger N-acetyl-l-cysteine could eliminate p-p38, p-ERK, and p-JNK upregulation (Huang et al., 2018; Fan et al., 2020). In summary, ROS can induce autophagy via the MAPK signaling pathway in bacterial infection. Accumulating evidence has suggested the essential functions of MAPK signaling in male fertility (Zhang and Lui, 2015; Ni et al., 2019). The p38 MAPK signaling regulates JAM-B expression via phosphorylating the ETS domain transcription factor, which is essential for the migration of germ cells (Wang and Lui, 2009). ERK signaling can interact with FGF-4 or GDNF to affect the self-renewal of spermatogonia stem cells (Yamamoto et al., 2000; Hasegawa et al., 2013). JNK signaling is associated with tight junctions and adherens junctions dynamics in testis. The activated JNK signaling increases ICAM-1 expression, which can stabilize tight junctions dynamics (De Cesaris et al., 1999).

#### NF-E2-Related Factor 2 Signaling in Oxidative Stress-Autophagy Axis

Several studies suggest that Nrf2 signaling affects male reproductive function. A direct evidence indicates that down-regulation of Nrf2 triggers spermatogenic cells ferroptosis. In turn, the activated Nrf2 increases busulfan-treated sperm motility and concentration (Zhao et al., 2020). It also has been shown above that Nrf2/Keap1/AREs signaling is a key pathway in oxidative stress. Most studies on oxidative stress-induced Nrf2 signaling have focused on autoimmune disease and tumorigenesis, but seldom relate to pathogenic microorganism infection (Zhang and Gordon, 2004; Gilardini Montani et al., 2019; Tian et al., 2020). A recent study reported that *Mycoplasma gallisepticum* could induce oxidative stress and activate Nrf2 signaling. On the one hand, activation of the Nrf2 pathway contributes to maintaining mitochondria’s normal functions, which is one of the key factors in maintaining sperm homeostasis during bacterial infections (Ishfaq et al., 2019). In addition, suppressing Nrf2 signal would decrease HO-1 expression, and subsequently result in ROS induction (Ko et al., 2016). On the other hand, many studies have found that the Nrf2 signal can increase phosphorylation of AMPK, ultimately inducing autophagy via suppressing the phosphorylation of mTORC1 and its related protein (Shen et al., 2020). Additionally, mitophagy and the Nrf2 signal are interdependent. Phosphorylation of the autophagy-adaptor protein p62 is related to persistent activation of Nrf2 (Ichimura et al., 2013). Thus, we hypothesize that there may be such a signaling pathway in the infection process. After the pathogenic microorganisms invade the body, the accumulation of ROS induces oxidative stress. Subsequently, Nrf2 dissociates from Keap1 and then heterodimerizes with Maf protein, which binds to the ARE motif of the P62 promoter. Finally, the increased expression of P62 induces mitophagy (Geisler et al., 2010). Mitophagy, in turn, can maintain mitochondrial homeostasis by eliminating excessive ROS together with damaged mitochondria (Ma et al., 2020).

#### Other Signaling in Oxidative Stress-Autophagy Axis

When the host is in a state of intense inflammation induced by bacterial infections, macrophages can produce a substantial amount of NO and O^2^−, which induce plenty of ROS and oxidative stress. Subsequently, ROS induces the HIF-1-mediated transcription of *BNIP3*, *BNIP3L*, and *NIX* genes. Their proteins further induce autophagy by competing with beclin-1 for binding BCL2 (Mahalingaiah and Singh, 2014). HIF-1 signaling-induced free beclin-1 can cause mitophagy, clearing the damaged mitochondria and decreasing ROS production (Xu Y. et al., 2020). Several inflammatory cytokines and ROS can be induced in bacterial LPS-exposed Leydig and Sertoli cells (Duan et al., 2016; Li et al., 2019). TNF-α and IL-6 are involved in regulating apoptosis of Sertoli cells (Yao et al., 2009; Zhang et al., 2014). Moderate ROS indirectly enhances the AMPK phosphorylation (for example, inducing mitochondrial ATP production) and then attenuates mTOR activation (Hinchy et al., 2018). Furthermore, the inhibition of mTOR signaling is a key factor in the autophagy phenomenon. Moreover, AMPK also exists in the midpiece of sperm (Calle-Guisado et al., 2017). AMPK activity helps maintain sperm mitochondrial membrane potential, which is also a key signal of mitophagy (Martin-Hidalgo et al., 2018). Additionally, the AMPK pathway affects Sertoli cells function. The activated AMPK signaling can induce glucose in rat Sertoli cells, which is a preferring energy source of spermatids (Kishimoto et al., 2015; Nguyen, 2017). AMPK activation also can inhibit the proliferation of rat Sertoli cells through suppression of mTOC1 (Ni et al., 2019). ROS-induced oxidative stress can also regulate the activation of PI3K signaling during bacterial infection (Mistry et al., 2019). PI3K signaling can also inhibit mTOR activation via AKT and GSK3B signals and promote ULK1 phosphorylation and transcription of multiple autophagy-related genes (Sciarretta et al., 2014; Guo et al., 2018). In addition, PI3K signaling takes part in regulating the proliferation of piglet Sertoli cells and testicular microcirculation homeostasis, but the precise mechanism is still unclear (Sun et al., 2015; Long et al., 2018). Furthermore, Pun’s research has shown that ROS inhibitors could prevent the LPS-induced SIRT1-FoxO3A axis, which involves transcription autophagy-related genes such as *LC3* and *BNIP3* (Pun et al., 2015). Oxidative stress-induced FoxO3A activation leads to the induction of autophagy (Li et al., 2015).

**FIGURE 1:**
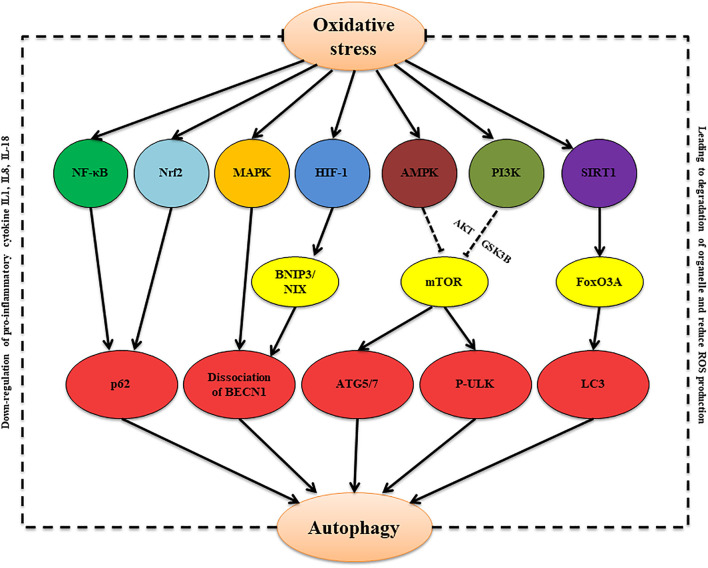
The proposed oxidative stress/autophagy cross-talk in the bacterial infection condition. Oxidative stress induces autophagy via multiple signaling pathways, including NOX- NF-κB-p62 signaling, Nrf2-Keap1-AREs signaling, MAPK-beclin-1 signaling, HIF-1-BNIP3/NIX-beclin-1 signaling, AMPK/PI3K-mTOR signaling, and SIRT1-FoxO3A-LC3/BNIP3 signaling. Autophagy, in turn, can attenuate oxidative through inducing degradation of damaged organelles and decreasing production of pro-inflammatory cytokines and ROS.

To sum up, during bacterial infection of the male reproductive system, oxidative stress can increase ROS levels. On the one hand, oxidative stress-damaged tissues and cells lead to increased ROS aggravating damage and imbalance of physiological homeostasis. On the other hand, ROS also can induce autophagy, which appears to be a key protective mechanism against oxidative stress and intracellular abnormalities (Table 2). Multiple signaling pathways, including NOX- NF-κB-p62 signaling, MAPK-beclin-1 signaling, HIF-1- BNIP3/NIX-beclin-1 signaling, AMPK/PI3K-mTOR signaling, SIRT1-FoxO3A-LC3/BNIP3 signaling, and Nrf2-Keap1-AREs signaling, are involved in the oxidative stress-autophagy axis (Figure 1). There are still few studies on how autophagy regulates oxidative stress. Several studies found that the regulation of autophagy on oxidative stress under different pathological conditions showed different results, and autophagy-mediated changes of production of cytokines and ROS and organelle degradation play key roles during these processes (Kim et al., 2007; Nakahira et al., 2011; Chen et al., 2015; Li Q. et al., 2018; Saxena et al., 2018). The specific mechanisms in these processes remain to be further studied.

**TABLE 2:** Key signaling molecules associated with oxidative stress-autophagy axis and their effect on male fertility.

Signaling molecules	Targeted tissue/cells	Involvement of male fertility	References
TGF-β3	Testis	Regulating the blood-testis barrier dynamics	Zhang and Lui, 2015
ATG5/7	Sertoli cells	Maintaining cytoskeletal organization of sertoli cells	Liu et al., 2016
FGF-4	Sertoli cells	Affecting the self-renewal of spermatogonia stem cells	Yamamoto et al., 2000
IL-6	Sertoli cells	Affecting blood-testis barrier integrity and proliferation of sertoli cells	Jenab and Morris, 1997; Zhang et al., 2014
Glucose	Sertoli cells	Maintaining sertoli cells function	Nguyen, 2017
mTOC1	Sertoli cells	Regulating proliferation of sertoli cells	Ni et al., 2019
PI3K	Sertoli cells, testis	Regulating proliferation of sertoli cell, affecting testis integrity	Sun et al., 2015; Long et al., 2018

## Conclusion

Bacterial infection in the male reproductive system is one of the key factors affecting male fertility. The main factors leading to male sterility are inflammation of the reproductive system, injury of the male reproductive organ, and spermatogenesis disorder induced by pathogenic bacterial infection. Furthermore, oxidative stress is closely related to these pathological processes. This review has focused on how oxidative stress forms during genital tract pathogen infections involving *E. coli*, *S. aureus*, *U. urealyticum*, *C. trachomatis*, *N. gonorrhoeae*, *S. agalactia*, and *S. saprophyticus*, and how oxidative stress induces inflammation, tissue damage, and spermatogenesis dysfunction. Furthermore, we describe Nrf2/Keap1/AREs, PI3K/Akt/mTOR, and TLR signaling as the main signal transduction pathways of oxidative stress during bacterial infections. Autophagy, which is always accompanied by oxidative, can help maintain host homeostasis. We discussed the effect of oxidative stress-autophagy interactions on male fertility in bacterial infection. MAPK, HIF-1, AMPK, PI3K, SIRT1, and Nrf2 are activated in response to oxidative stress, and sequentially, they can invoke autophagy by regulating beclin-1, mTOR, FoxO3A, and p62. Autophagy, in turn, can affect the secretion of pro-inflammatory cytokines, degradation of organelle and production of ROS.

Since oxidative stress and autophagy both serve dual functions in eliminating pathogenic, there is a very promising question of how to use oxidative stress and autophagy to maintain male reproductive vitality. Here we put forward the following thinking. First, it is important to understand the infectious properties of different bacteria. For bacteria that can be cleared by autophagy, we can use autophagy inducers to maintain male reproductive vitality. For bacteria that can use autophagy to achieve immune escape and help them survive, we can use autophagy inhibitors to maintain male reproductive vitality. Since excessive oxidative stress can cause tissue and organ damage, we also need to pay attention to controlling levels of oxidative stress. Secondly, according to our summary of signals affecting both oxidation and autophagy, we can further look for potential targets that affect spermatogenesis, sperm quality and inflammation. Therefore, this review can also provide a reference for treating bacterial infection of the male reproductive system from the perspective of oxidative stress-autophagy. In short, the mutual regulation and restriction of oxidative stress and autophagy guarantee the elimination of pathogenic bacteria and the balance of physiological homeostasis in male reproductive organs.

## Author Contributions

SD and SW conceptualized this manuscript. SW and KZ wrote this manuscript. YY assisted with the edited version. SW and JL acquired funding. All authors contributed to the article and approved the submitted version.

## Conflict of Interest

The authors declare that the research was conducted in the absence of any commercial or financial relationships that could be construed as a potential conflict of interest.

## Publisher’s Note

All claims expressed in this article are solely those of the authors and do not necessarily represent those of their affiliated organizations, or those of the publisher, the editors and the reviewers. Any product that may be evaluated in this article, or claim that may be made by its manufacturer, is not guaranteed or endorsed by the publisher.
